# The Potential of Iodized Cadaveric Thumb Fingerprints as Markers of Perfusion Completion During Embalming: An Experimental Observational Study

**DOI:** 10.7759/cureus.83772

**Published:** 2025-05-09

**Authors:** S Surraj, Mrudula Chandrupatla

**Affiliations:** 1 Department of Anatomy, All India Institute of Medical Sciences (AIIMS) Hyderabad, Hyderabad, IND

**Keywords:** arterial embalming, color change, iodine, reduced valency, tissues

## Abstract

Background and aim: The formalin-based method of embalming is the most common procedure employed for the preservation of cadavers in most medical schools of South Asia. The adequacy of perfusion of formalin through the cadaveric tissues would aptly mark the time of completion of embalming. However, there is currently no objective evidence to justify the endpoint of perfusion in embalming. This study addressed the aforementioned problem by utilizing cadaveric thumb fingerprints coated with a modified iodine-based solution that indicated the completion of perfusion during embalming.

Methods: Cadaveric thumb fingerprints and thumb nail plates coated with a modified solution of iodine offered a medium wherein the color changes of perfusion were observed from pale brown to colorless.

Results: The color changes at the fingerprints and nail plates coincided with the onset of lip stiffness and confirmed the completion of perfusion based on their consistency in all the cadavers.

Conclusions: The iodine-based method of color change could definitely serve as a marker of perfusion during embalming.

## Introduction

The procedure of arterial embalming using formalin-mixed embalming fluid is usually employed to preserve the cadaver for longer periods of time for the purpose of scientific dissections. This procedure ought to be stopped at the right time to avoid the problem of under-fixation of tissues, thereby reducing the chances of putrefaction [1-3]. Simultaneously, the procedure should not be delayed, lest the tissues harden excessively. Hence, the perfusion of formalin through the tissues serves as the key to helping the anatomists decide the time to stop this procedure at the right time [2,3]. To date, there have been varied opinions among anatomists to deduce the signs of adequacy of tissue perfusion that would mark the completion of embalming and four such external signs have been speculated to date, which include abdominal fullness, increased skin turgor, frothy ooze through the orifices, and lip stiffness, without any evidence to objectively support these claims [2-4]. The interpretation of these signs is subjective and fraught with multiple speculations [3-5]. This study addressed that problem by using an experimental approach to deduce the adequacy of perfusion of tissues through formalin by making it react with a modified iodine solution at the fingernail plates (where diffusion of formalin would occur rapidly due to the minute pores found within the tough lattices of keratin that would facilitate easy seepage and perfusion of formalin) and at the point of contact of fingerprints (where color changes in iodine would be pronounced visibly on paper). This enabled a clearer understanding of color changes in perfusion in order to aptly decide the time of completion of embalming [5-8].

## Materials and methods

This was an experimental observational study conducted on 10 freshly procured adult cadavers whose age range was between 50 and 60 years. This research was conducted in a cadaveric anatomy lab, wherein an inert environment was provided by supplementing the lab with activated charcoal that helped to absorb the excessive formalin vapors, excess humidity, and excess moisture, while also regulating an even temperature in the lab. The lab temperature was maintained at 28°C for each of the cadavers that were embalmed. The mean weight of the cadavers was 65.20 kg, with the maximum weight being 68.21 kg and the minimum weight being 61.10 kg.

This study included only those cadavers that were obtained through willful body donations and after obtaining clearance from the local police, local medical officer, and/or district magistrate. This study excluded those cadavers with chronic viral diseases, lacerations, amputated limbs, or skin excoriations. Cadavers with putrefaction, early-onset decomposition, and/or maggots were also excluded from the study. The cadavers were subjected to an arterial embalming procedure within two hours of procurement into the lab and within five hours of death [5-7]. This study was conducted between September 2024 and March 2025.

**Figure 1:**
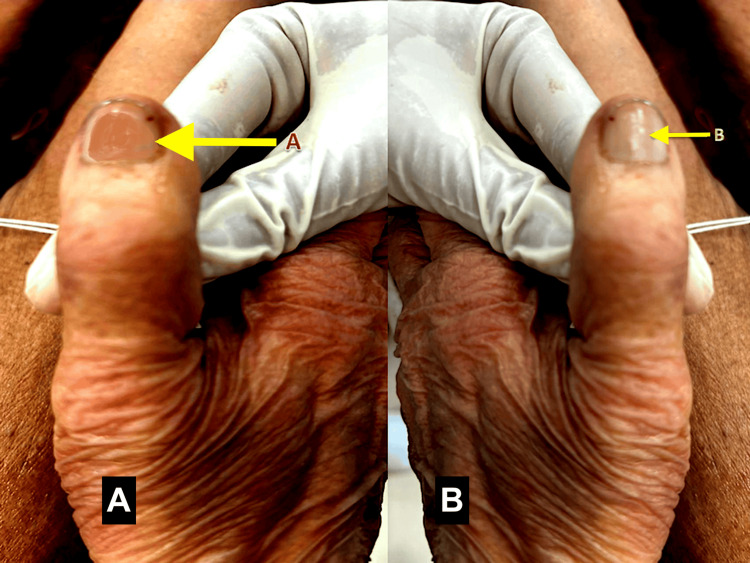
Iodine changes over the nail plate during the embalming process (A) Before the start of embalming: a modified iodine solution is applied to the nail plate before embalming (yellow arrow). (B) Upon the completion of tissue perfusion during embalming: the nail plate loses color on perfusion with formalin at the completion of embalming (yellow arrow). Original image by the authors.

Prior to the procedure of formalin-based embalming, the left and right thumbnail plates of the cadavers were coated with 2 ml of modified iodine solution. This solution was prepared by mixing 2% povidone iodine solution (dark brown in color) with 5 mg of detergent washing powder (which contained equal proportions of sodium carbonate and sodium bicarbonate in molar volumes) in a petri dish [8,9]. The resultant solution mixture was known as modified iodine solution, which was pale brown in color, and this solution was applied to the left and right thumbnail plates using a paintbrush (Figure 1A). This was done in order to observe the color changes in the iodine-coated nail plates once the process of formalin-based perfusion had set in (Figure 1B).

**Figure 2:**
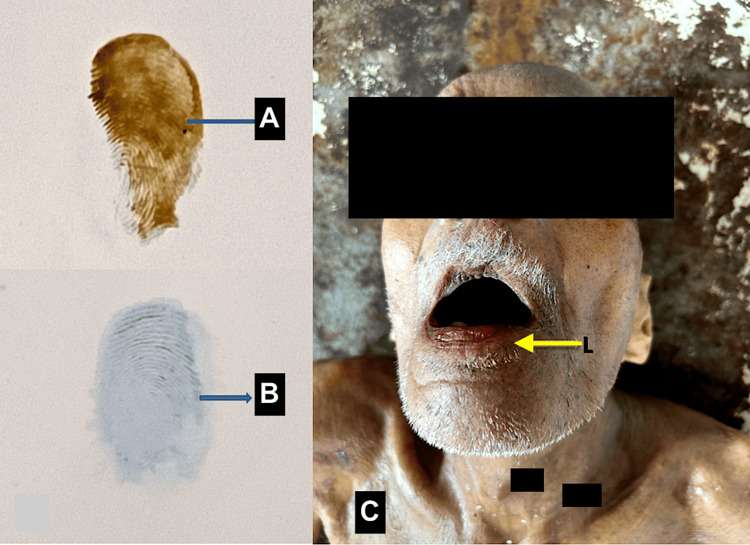
Color changes in the left thumb fingerprint and the onset of lip stiffness at the completion of embalming The sequential evolution of color changes in the left thumb fingerprint of a cadaver due to the reaction of formalin with reduced iodine showed (A) a clogged pale brown fingerprint at the start of embalming and (B) a colorless fingerprint following formalin perfusion, and embalming was then stopped. (C) Onset of lip stiffness as a sign of formalin perfusion during embalming in the cadaver (yellow arrow). Original image by the authors.

The aforementioned solution was also sprayed over the palmar surface of the distal phalanges of the thumb, and impressions were obtained from them on a white paper, which appeared as pale brown fingerprints prior to the start of embalming (Figure 2). The modified iodine solution was used instead of normal iodine for observing the color changes in fingerprints, as only this specific solution containing reduced carbonate ions would undergo titrational reactions and produce color changes upon contact with formalin, whereas the routinely used normal iodine solution would not be able to produce any color change upon coming into contact with formalin.

Prior to the start of embalming, 8000 ml of formalin-based embalming fluid (as available from the data records of the lab and as used routinely for embalming all the cadavers prior to the start of this study based on the standardization of results of our anatomy cadaveric lab) was filled in the graduated jars of the motorized embalming apparatus [6-9]. The mechanized pump-based arterial method of scientific embalming was used in all of the cadavers at a speed of 0.15 L/minute and at a pressure rate of 4 oz/minute. The femoral arterial route was used for this motorized embalming procedure.

Once the arterial embalming was started, the time of onset of embalming was noted, and the four subjective signs of perfusion mentioned in the literature were observed: frothy orificial ooze, abdominal fullness, skin turgor, and lip stiffness. Their respective times of onset were also noted [2-5]. Immediately following the onset of color changes from pale brown to colorless at the nail plates, the thumb fingerprint impressions were taken immediately and found to be colorless. The embalming procedure was then stopped immediately, irrespective of the onset of other subjective signs of perfusion. The findings were noted, compared, and then analyzed. Relevant photographs of the color changes were taken. This study was performed only after obtaining appropriate clearance from the All India Institute of Medical Sciences (AIIMS), Bibinagar, Hyderabad, India's research council, and subsequent ethical clearance.

## Results

The changes in the color of the thumbnail plates from pale brown (before embalming) to colorless (after perfusion through formalin) were observed and photographed (Figure 1). The weight of the cadavers did not have any impact on the iodine-based color changes in fingerprints. Similarly, the color changes in the thumb fingerprints were also observed and photographed (Figure 2). The embalming procedure was stopped at the onset of the aforementioned color changes, as it indicated strong visible evidence of formalin seepage and perfusion through the tissues upon coming into contact with the modified iodine solution in each cadaver. Incidentally, the onset of lip stiffness also coincided with the color changes of iodine at the nails and fingerprints in each cadaver (Figure 2).

The time of onset of the speculated subjective signs of formalin perfusion, namely, skin turgor, abdominal fullness, frothy orificial ooze, and lip stiffness, was noted by three independent observers, and the mean time of onset of each of these signs in all the cadavers was noted. Similarly, the mean time of onset of color changes in the thumbnail plates and thumb fingerprints was also noted, and the results were tabulated (Table 1).

**Table 1:** Sequential mean time of onset of the signs of perfusion of formalin in the cadavers SD: standard deviation

Cadaver number	Mean (+/- SD) time of onset of skin turgor (sec)	Mean (+/- SD) time of onset of abdominal fullness (sec)	Mean (+/- SD) time of onset of frothy orificial ooze (sec)	Mean (+/- SD) time of onset of lip stiffness (sec)	Mean (+/- SD) time of onset of color change at thumbnail plates (sec)	Mean (+/- SD) time of onset of color change in thumb fingerprints (sec)
1	600 +/- 0.2	650 +/- 0.2	800 +/- 0.2	900 +/- 0.2	910 +/- 0.3	910 +/- 0.2
2	650 +/- 0.3	700 +/- 0.3	810 +/- 0.3	920 +/- 0.2	900 +/- 0.3	910 +/- 0.2
3	660 +/- 0.2	750 +/- 0.4	800 +/- 0.3	900 +/- 0.2	900 +/- 0.3	900 +/- 0.3
4	600 +/- 0.2	700 +/- 0.4	820 +/- 0.3	910 +/- 0.2	900 +/- 0.3	900 +/- 0.3
5	620 +/- 0.2	750 +/- 0.4	840 +/- 0.4	900 +/- 0.2	900 +/- 0.3	900 +/- 0.3
6	700 +/- 0.3	760 +/- 0.3	800 +/- 0.3	920 +/- 0.2	900 +/- 0.3	910 +/- 0.3
7	600 +/- 0.4	740 +/- 0.2	850 +/- 0.3	920 +/- 0.2	900 +/- 0.3	900 +/- 0.3
8	620 +/- 0.2	740 +/- 0.4	850 +/- 0.3	900 +/- 0.2	910 +/- 0.3	900 +/- 0.3
9	660 +/- 0.2	750 +/- 0.4	850 +/- 0.3	900 +/- 0.2	900 +/- 0.2	900 +/- 0.2
10	630 +/- 0.2	700 +/- 0.4	800 +/- 0.3	900 +/- 0.4	900 +/- 0.2	900 +/- 0.2

Table 1 shows that the onset of lip stiffness coincided with the onset of color changes in the nails and fingerprints of each cadaver, strongly suggesting the evidence of complete perfusion of formalin at this point in time.

The mean time difference between the start of the embalming procedure and the onset of color changes in the fingerprints was noted for all the cadavers and subsequently compared with the mean volume of utilized embalming fluid (as observed by three independent observers) using the paired t-test, given their interdependence as variables. A positive association was obtained between them, objectifying the evidence obtained through color changes of formalin perfusion on reacting with the reduced state of iodine (Table 2).

**Table 2:** Comparison between the mean time difference (seconds) between the start and end of the embalming procedure marked by the appearance of color change from brown to colorless at the thumb fingerprints and the mean volume of used embalming fluid (ml) The p-value was assessed using Student's t-test for a 95% double-sided confidence interval. SD: standard deviation

Cadaver number	Mean (+/-SD) time difference between the start and end of the embalming procedure marked by the appearance of color change from brown to colorless at the thumb fingerprints (sec)	Mean (+/-SD) volume of used embalming fluid (ml)	p-value (for a double-sided 95% confidence interval)
1	910 +/- 0.2	3500 +/- 0.31	0.0001
2	910 +/- 0.2	3500 +/- 0.21	
3	900 +/- 0.3	3600 +/- 0.31	
4	900 +/- 0.3	3600 +/- 0.32	
5	900 +/- 0.3	3500 +/- 0.21	
6	910 +/- 0.3	3500 +/- 0.20	
7	900 +/- 0.3	3700 +/- 0.20	
8	900 +/- 0.3	3500 +/- 0.30	
9	900 +/- 0.2	3500 +/- 0.20	
10	900 +/- 0.2	3600 +/- 0.30	

Additionally, the volumes of formalin-based embalming fluid remaining in the jar at the end of the procedure were noted by three independent observers (two of them being the researchers of this study and another one being the lab technician). Their mean values were obtained for each cadaver and associated with the time of onset of color change in the fingerprints. The strong positive association that was found between the mean time of onset of fingerprints and the mean volume of embalming fluid (ml) remaining in the jar at the end of the embalming procedure suggested that the termination of the embalming procedure was largely dependent on the completeness and adequacy of formalin seepage and perfusion through the tissues. The mean volume of embalming fluid was determined by deducing the mean of the observed values of residual embalming fluid by the three independent observers to avoid bias. The inter-observer reliability among the three observers' findings was assessed using the intra-class correlation coefficient (ICC) that was estimated using the GraphPad Prism software version 2 (GraphPad Software, Inc., La Jolla, California). The ICC was found to be 0.822 between the observers' findings, indicating a robust measure of inter-observer reliability.

## Discussion

The transparency of nail plates would adequately reveal the intensity of color changes due to perfusional chemical reactions between molecules when compound mixtures are applied, as the pores present between their lattices of keratin would easily suck the fluid that gets percolated from the nail matrix due to a reflex syphoning effect [2]. Fingerprints, on the other hand, contain pores from sweat gland ducts distributed over their loops that get clogged upon coming into contact with formalin due to the crosslinking and precipitation of proteins, thereby rendering the color changes over the fingerprints remarkably noticeable [2,9]. Consequently, these two areas served as phenomenal sites of color change, marking the completion of perfusion with formalin and directing the researchers to stop the embalming procedure at the right time without wasting excess formalin.

The onset of skin turgor, the appearance of abdominal fullness, the occurrence of frothy ooze through the cadaver's orifices, and the onset of lip stiffness are just a few of the subjective signs that indicate the completion of embalming through the percolation and seepage of formalin into the cadaveric tissues [2-4]. Different embalming personnel may interpret these signs differently. Of the aforementioned signs, this study's findings showed that the beginning of lip stiffness corresponded with the beginning of a color change at the fingernail plates covered with modified iodine solution. This might be because, in contrast to the other locations, the lips' soft and muscular architecture allowed for a distinct, visible reaction to the penetrating effects of formalin [5-8].

**Figure 3:**
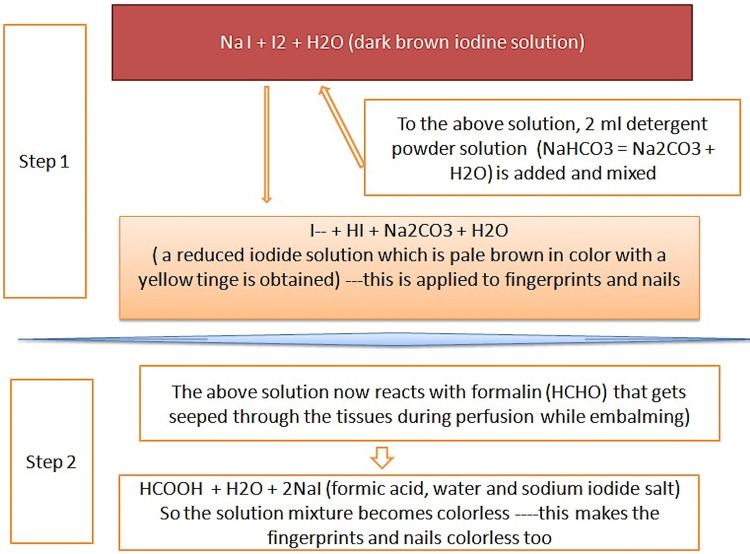
The phenomenon of the appearance of color change in modified iodine solution Image created by the authors.

Since the time of onset of color change in the fingerprints and the fingernail plates coincided with the time of onset of stiffness of the lips, this could possibly denote that the phenomenon of cadaveric tissue perfusion during embalming could be a fairly uniform process with a rapid, even circulation of formalin followed by its equal distribution to all body parts at the same time period. Environmental factors could not play a role in the results of color changes in fingerprints because an inert environment had been maintained in the lab. This negates the view already opined in previous postmortem studies that mechanical kinks within the body due to arterio-capillary-venous bends may impede the equal rate of formalin perfusion [5,8,9]. Since the other subjective signs, such as abdominal fullness, skin turgor, and frothy orificial ooze, preceded the onset of color change at the nail plates and fingerprints as observed in this study, the authors would like to opine that these subjective signs might not be sufficient to provide evidence for tissue perfusion during embalming as compared to the pronounced, visibly marked color changes due to the reaction between formalin and reduced iodide solution over the nail plates and fingerprints, as illustrated in Figure 3.

The povidone iodine depicted in the first step of the equation comprised sodium iodide and molecular iodine, which were originally dark brown in color. This solution turned pale brown when mixed with detergent powder made of sodium bicarbonate and sodium carbonate [8]. The pale brown color occurred due to the formation of a reduction substrate that occurred in the solution wherein the molecular state of iodine was reduced to its iodide form, resulting in the separation of free radicals of iodine that, in turn, floated in a pool of hydrogen iodide and sodium carbonate admixed with water. The resultant solution that was formed was known as a modified iodine-based solution, and it served as a titrational indicator of color change on coming into contact with perfused formalin [5-7,9]. The second step of the equation revealed that the modified iodine solution, on reacting with perfused formalin through the tissue spaces, became colorless due to the formation of sodium iodide and formic acid [9,10]. This second step denoted the end point of perfusion as seen in the cadavers with a visible transition in color change, at which point the procedure of arterial embalming was terminated.

Limitations

Since this is an experimental pilot study done to validate the effects of iodine-based fingerprints in cadavers, the sample size was limited. The limited availability of cadavers in this part of the country also played a role in the limited sample size due to the paucity of voluntarily available body donors who agreed to willfully donate their bodies after death. The toenail plates and other palm print impressions could not be obtained or assessed properly due to the onset of rigor mortis in those areas; this could also be considered a limitation of this study.

## Conclusions

Fingerprint color changes based on modified iodine solution helped to save the excessive irritant exposure to formalin, as the adequacy of perfusion could be easily determined using a modified iodine-based solution as an indicator to objectify the completion of embalming through a color change from pale brown to colorless at specific sites on the cadaver, especially using the fingerprints. This would not only reduce the time duration of the procedure but also simplify the same. It would also help save the wastage of formalin-based embalming fluid and reduce the burden of excessive purchase costs.
